# DOES IMPROVING SLEEP IMPROVE COGNITION IN OLDER INDIVIDUALS WITH INSOMNIA?

**DOI:** 10.1093/geroni/igz038.173

**Published:** 2019-11-08

**Authors:** Christina McCrae

**Affiliations:** University of Missouri-Columbia, Columbia, Missouri, United States

## Abstract

Late life insomnia is associated with worse cognitive performance. Behavioral/cognitive behavioral treatments for insomnia (BBT-I, CBT-I) improve sleep in older adults, but findings are mixed for cognition. This presentation examines the effects BBT-I and CBT-I on sleep and cognition across three RCTs involving older individuals (community-dwelling [N=62, Mage=69.45(SD=7.71)], chronic pain [N=64, Mage=53.2 (SD=13.7)], dementia caregiving [N=36, Mage=62.32 (SD=6.71]). Sleep was assessed using daily diaries and actigraphy for 1-2 weeks prior to randomization to treatment or control. Cognition was measured using standardized executive functioning, memory, and attention measures. Multiple regressions revealed improved executive functioning following treatment (caregivers), associations between improved executive performance and greater pain/sleep improvements (chronic pain), and associations between improved attention and processing speed and improved sleep 9-months following treatment (community-dwelling). BBT-I/CBT-I hold promise for improving cognition in older aged individuals with insomnia. Research is needed to determine what factors influence/which patients are most likely to experience cognitive benefits.

